# Lipase-Responsive Amphotericin B Loaded PCL Nanoparticles for Antifungal Therapies

**DOI:** 10.3390/nano13010155

**Published:** 2022-12-29

**Authors:** Evelyn Osehontue Uroro, Richard Bright, Andrew Hayles, Krasimir Vasilev

**Affiliations:** 1 UniSA STEM, University of South Australia, Mawson Lakes, SA 5095, Australia; 2 College of Medicine and Public Health, Flinders University, Bedford Park, SA 5042, Australia

**Keywords:** amphotericin B, polycaprolactone (PCL), lipase, drug delivery, cytocompatible, antifungal activity

## Abstract

Amphotericin B is an antifungal drug used for the treatment of invasive fungal infections. However, its clinical use is limited due to its serious side effects, such as renal and cardiovascular toxicity. Furthermore, amphotericin B is administered in high doses due to its poor water solubility. Hence, it is necessary to develop an on-demand release strategy for the delivery of amphotericin B to reduce cytotoxicity. The present report describes a novel encapsulation of amphotericin B into lipase-sensitive polycaprolactone to form a nanocomposite. Nanocomposites were produced by the oil-in-water method and their physicochemical properties such as size, hydrodynamic diameter, drug loading, and zeta potential were determined. The in vitro release of amphotericin B was characterized in the presence and absence of lipase. The antifungal activity of the nanocomposites was verified against lipase-secreting *Candida albicans*, and cytotoxicity was tested against primary human dermal fibroblasts. In the absence of lipase, the release of amphotericin B from the nanocomposites was minimal. However, in the presence of lipase, an enzyme that is abundant at infection sites, a fungicidal concentration of amphotericin B was released from the nanocomposites. The antifungal activity of the nanocomposites showed an enhanced effect against the lipase-secreting fungus, *Candida albicans*, in comparison to the free drug at the same concentration. Furthermore, nanoencapsulation significantly reduced amphotericin B-related cytotoxicity compared to the free drug. The synthesized nanocomposites can serve as a potent carrier for the responsive delivery of amphotericin B in antifungal applications.

## 1. Introduction

Fungal infections are a major healthcare burden, causing an estimated 2 million global deaths annually [1,2,3]. One of the most prominent fungal species capable of causing life-threatening infections is *Candida albicans.* While it frequently resides as a commensal species on the skin and mucosal tissue, it is also an opportunistic pathogen that can cause deep tissue infection and systemic invasion [4]. Superficial infections can occur in the mouth and vagina, causing thrush symptoms, while systemic infections can arise from invasion into the bloodstream and dissemination to internal organs, the latter being frequently life-threatening [4,5]. Some antifungal agents used in clinical treatments include azoles (e.g., clotrimazole and fluconazole), echinocandins (e.g., caspofungin and micafungin), and polyenes (e.g., nystatin and amphotericin B (AMB)) [6]. Fungi share many similarities with mammalian cells. Due to these similarities, most antifungal drugs are also cytotoxic thus, non-selective antifungal therapy could lead to mammalian cell toxicity. For example, AMB interacts with cholesterol in mammalian cell membranes, and as a result, the drug can cause nephrotoxicity and cardiotoxicity [7,8]. The antifungal mechanism of action of AMB involves the interaction of the drug with the ergosterol in the fungal cell membrane, thereby altering their cell structures and permeability [7,9]. Due to its poor water solubility and bioavailability, AMB is required to be administered in a high dose [10]. Additionally, the overuse of antifungal drugs and the emergence of drug-resistant fungi have greatly challenged antifungal therapies [11,12,13]. 

Some factors that influence the virulence and pathogenic capacity of various fungal species, such as *Candida albicans,* are their dimorphic growth and secretion of hydrolytic enzymes such as proteinases and lipases [14]. Rationally designed delivery platforms can turn the strength of a pathogen into its weakness. For instance, the presence of lipases at infection sites can be utilized in an on-demand strategy for the triggered delivery of antifungal drugs [11]. Polycaprolactone (PCL) has been widely used for designing drug-delivery platforms due to its biocompatibility and the fact that the polymer has already been approved by regulatory bodies for biomedical applications [15]. It is also well known that PCL is susceptible to degradation by lipases. The latter provides an opportunity to devise an on-demand delivery vehicle for AMB that is triggered by lipase-secreting fungal pathogens.

This work aimed to formulate a nanoscale on-demand delivery platform utilizing PCL as a carrier for AMB. The method of preparation of polymeric nanoparticles is highly dependent on the solubility of the drug intended for encapsulation. In encapsulating various hydrophobic drugs, emulsification techniques using emulsifiers such as polyvinyl alcohol (PVA) are mostly employed, and the nanoparticles are developed using evaporation techniques [16]. Drugs with poor water solubility, including AMB, can only be encapsulated in hydrophobic polymers using specific techniques as this would have a significant impact on the encapsulation efficiency. Considerable research has focused on the encapsulation and delivery of AMB using polymeric nanoparticles, such as poly(lactide-co-glycolide) (PLGA) [17,18,19]. However, these strategies lead to indiscriminate drug release, often delivering concentrations well below or well above those needed [20,21]. The former is insufficient to eliminate infection but creates an environment that promotes the development of resistance. The second can cause considerable toxicity which is also a major concern in antifungal therapy [22]. On-demand delivery and release platforms for antifungal agents are considered a way forward to treat fungal infections effectively. This can be achieved by using appropriate techniques to encapsulate antifungals in stimuli-responsive materials [7]. Herein, we report the synthesis of PCL nanoparticles for the lipase-triggered release of AMB and demonstrate their responsiveness to fungal lipases, efficacy in killing clinically relevant fungal species, and cytocompatibility with primary human dermal fibroblasts. 

## 2. Materials and Methods

### 2.1. Materials

AMB from *Streptomyces* sp. (~80% HPLC powder), PCL (M_w_ = 45,000 Da), PVA (M_w_ = 9000–10,000 Da, 80% hydrolysed) and dichloromethane (DCM) (CH_2_Cl_2_, M_w_ = 84.94 g/mol) were purchased from Sigma-Aldrich, MO, USA. Methanol and dimethyl sulfoxide (DMSO) were purchased from Chem-Supply, SA, Australia. Phosphate buffered saline (10 mM phosphate, 150 mM sodium chloride) tablets were purchased from Thermo Fisher Scientific, Waltham, MA, USA. Lipase from *Candida rugosa* (lipase activity ≥ 700 U/mg) was obtained from Sigma-Aldrich, NSW, Australia. Dulbecco’s Modified Eagle Medium (DMEM) was purchased from Gibco Life Technologies, MA, USA, and prepared according to the manufacturer’s instructions. Ultrapure Milli-Q (MQ) water with a resistivity of 18.2 mΩ (Millipore, Burlington, MA, USA) was used in all synthesis and preparations unless otherwise stated.

### 2.2. Synthesis of AMB Loaded Nanoparticles (PCL-AMB NPs)

Synthesis of PCL-AMB NPs was carried out using a modified emulsion solvent method [23]. Briefly, 20 mg of AMB was dissolved in 0.8 mL of DMSO. After complete dissolution, 3.2 mL of methanol was added to the solution to make up 4 mL. The mixture was vortexed for proper mixing and set aside. To obtain the polymeric solution, 300 mg of PCL pellets were dissolved in 6 mL DCM and the entire solution was added to the 4 mL of AMB solution and vortexed for 5 min. The mixture was completely added dropwise to 40 mL of 1% PVA prepared in MQ water, stirring at 500 rpm before sonication using a Branson 450 Sonicator (VWR, PA, USA) for 3 min on ice. The emulsion was left to stir overnight, to allow sufficient evaporation of the organic solvents. After overnight stirring, the nanoparticles were collected by ultra-centrifugation at 23,200× *g* for 30 min at 4 °C using a Thermo Scientific™ Sorvall™ RC 6 Plus Centrifuge (Thermo Fisher Scientific, MA, USA). Nanoparticles were washed three times with MQ water before lyophilization using a ModulyoD freeze dryer (Thermo Fisher Scientific, MA, USA) for 72 h and stored at 4 °C for further use. Similarly, unloaded, or blank nanoparticles (PCL NPs) were prepared in DMSO and methanol solutions, with PVA as the emulsifier. Blank nanoparticles were stored similarly to PCL-AMB NPs.

### 2.3. Physicochemical Characterization of Nanoparticles 

Fourier transform infrared (FTIR) spectroscopy was carried out using a PerkinElmer Spectrum 100 FT-IR spectrometer (ATR spectrum, PerkinElmer, MA, USA). Scans were performed in the wavelength range between 4000 and 450 cm^−1^. Plots were obtained using the PerkinElmer Spectrum IR software (version 10.6.1.942). The UV-Vis spectra of PCL NPs, AMB, and PCL-AMB NPs were obtained using a Shimadzu UV-3600 UV-Vis-NIR Spectrophotometer (Shimadzu Corporation, Kyoto, Japan). Zeta potential and particle sizes were obtained using a Malvern Instruments Zetasizer Nano ZS (Malvern Analytical, Malvern, UK). 

PCL-AMB NPs and PCL NPs surface morphologies were acquired using a Zeiss Merlin FEG Scanning electron microscope (Carl Zeiss, Jena, Germany). Briefly, lyophilized nanoparticles were deposited on a clean silicon wafer mounted on SEM stubs. Samples were sputter coated with 3 nm of platinum using a resolution sputter coater (Agar Scientific Ltd, Essex, UK) under an argon atmosphere before imaging at 2 kV.

The internal morphology of the nanoparticles was investigated using transmission electron microscopy (TEM, JEOL Ltd., Tokyo, Japan). Briefly, the aqueous solutions of lyophilized nanoparticles were placed on a copper grid covered with carbon film. The samples were left to dry at room temperature before imaging using a JEOL JEM-2100F-HR TEM at 80 kV.

Differential scanning calorimetry (DSC) was carried out on PCL-AMB NPs, PCL NPs, and AMB using a Waters DSC (Waters, DE, USA). Experiments were carried out in a nitrogen atmosphere using a sealed aluminium pan containing 2 mg of the samples, and an empty sealed aluminium pan was used as the reference. PCL NPs and PCL-AMB NPs were heated from ~40 °C to ~220 °C, cooled to ~25 °C and then heated a second time to ~250 °C at the rate of 10 °C/min. AMB was heated from ~25 °C to ~220 °C, cooled to ~25 °C and then heated the second time to ~250 °C at the rate of 10 °C/min. The obtained DSC data were processed using TA instruments (Universal Analysis 2000, Waters LLC), Version 4.5A.

### 2.4. Quantification of AMB by High-Performance Liquid Chromatography (HPLC)

High-performance liquid chromatography was carried out to determine the concentration of AMB in PCL-AMB NPs samples using an Agilent 1200 Liquid Chromatograph—1200 FLD/DAD (Agilent Technologies, Waldbronn, Germany). The column used was a Kinetex^®^ 5 µm C18 100 A, 250 × 4.6 mm, with C18 (ODS, octadecyl) 4 mm × 3 mm column guard (Phenomenex, NSW, Australia). The injection volume varied from 2 to 20 µL. The mobile solvent A was water, and solvent B was acetonitrile, with a flow rate set at 1.2 mL/min with the column temperature set to 25 °C. The diode array detector was conducted with absorbance measured at 415 nm.

### 2.5. Drug Loading and Encapsulation Efficiency

To determine the drug loading of PCL-AMB NPs, 1 mg/mL of lyophilized sample was dissolved in DMSO, followed by 10-fold dilution in DSMO. The sample was vortexed and then filtered using a 0.45 µm filter before measuring the absorbance at 415 nm. The concentration of AMB in the sample was then calculated using the calibration curve. Pure AMB was first dissolved in DMSO (2, 4, 6, 8, and 10 µg/mL) and quantified using a Shimadzu UV-3600 UV-Vis-NIR Spectrophotometer (Shimadzu Corporation, Kyoto, Japan) to obtain a calibration curve. The concentration of AMB in the sample was then calculated using the calibration curve. Drug loading was calculated using Equation (1). The yield and encapsulation efficiency of PCL-AMB NPs were obtained using Equations (2) and (3), respectively.

(1) $$\mathrm{Drug}\;\mathrm{loading}\;\left(\%\right)=\frac{\mathrm{Mass}\;\mathrm{of}\;\mathrm{loaded}\;\mathrm{drug}\;\mathrm{in}\;\mathrm{polymer}}{\mathrm{Mass}\;\mathrm{of}\;\mathrm{polymer}\;\mathrm{containing}\;\mathrm{drug}}\times 100$$

(2) $$\mathrm{Yield}\;\left(\%\right)=\frac{\mathrm{Mass}\;\mathrm{of}\;\mathrm{nanoparticles}\;\mathrm{obtained}}{\mathrm{Mass}\;\mathrm{of}\;\mathrm{drug}\;\mathrm{added}+\mathrm{mass}\;\mathrm{of}\;\mathrm{polymer}\;\mathrm{added}}\times 100$$

(3) $$\mathrm{Encapsulation}\;\mathrm{efficiency}\;\left(\%\right)=\frac{\mathrm{Total}\;\mathrm{mass}\;\mathrm{of}\;\mathrm{drug}\;\mathrm{in}\;\mathrm{nanoparticles}}{\mathrm{Mass}\;\mathrm{of}\;\mathrm{drug}\;\mathrm{added}}\times 100$$

### 2.6. In Vitro Drug Release Assay

In vitro drug release was carried out in the presence of 1 mg/mL lipase solution in 10 mM phosphate-buffered saline (PBS) pH 7.4 and the absence of lipase. Briefly, 5 mg of PCL-AMB NPs was placed in a 10 mL tube containing 3 mL of 10 mM PBS (pH 7.4). The tube was then placed on an orbital shaker (Ratek Platform mixer, Vic. Australia) at 100 rpm at 37 °C for 0, 0.5, 1, 2, 3, 4, 5, 7, 24, 48, and 72 h. The samples were centrifuged at 1778× *g* for 5 min for all time points, using a Sigma 1–15 K centrifuge (Sigma Laborzentrifugen, Osterode, Germany) and 0.5 mL of the supernatant was collected for HPLC analysis, as outlined above. The release kinetics were determined for PCL-AMB NPs in the presence and absence of lipase.

### 2.7. In Vitro Antifungal Activity of PCL-AMB NPs

#### 2.7.1. Culturing of *Candida albicans (C. albicans)*

*C. albicans* ATCC 10231, an opportunistic pathogen was used in this study due to its lipase-producing activity reported in the literature [24,25,26,27,28]. *C. albicans* was plated onto potato dextrose agar (Oxoid, ThermoFisher, MA, USA) and incubated overnight at 37 °C. Single colonies were selected and inoculated in 15 mL potato dextrose broth (Oxoid, ThermoFisher, MA, USA) and incubated overnight at 37 °C.

#### 2.7.2. Minimum Inhibitory Concentration (MIC) Assay

The MIC assay was carried out based on clinical and laboratory standard guidelines recommended by the National Committee for Clinical Laboratory Standards (NCCLS) [29]. Due to the low solubility of AMB, a working stock of 2 mg/mL AMB was dissolved in DMSO. MQ water was then added to achieve a solution with a concentration of 0.5% DMSO. Briefly, 100 µL of potato dextrose broth was aliquoted into a 96-well plate, followed by the addition of 100 µL of AMB (10 µg/mL in 0.5% DMSO) into the first well. Next, the wells were mixed and serially diluted (1:1) down to the last well. After that, 20 µL of *C. albicans*, 2.5 × 10^6^ CFU/mL was inoculated into wells. To determine the MIC of PCL-AMB NPs, 100 µL of potato dextrose broth was aliquoted into a 96-well plate, followed by 100 µL of PCL-AMB NPs to the first well, then serially diluted across the plate. Lastly, 20 µL of *C. albicans*, at a concentration of 2.5 × 10^6^ CFU/mL, was added to each well. Wells devoid of AMB or PCL-AMB NPs were used as positive controls. Plates were incubated at 37 °C for 24 h, and the optical density (OD_600_) was measured using a Biotek microplate spectrophotometer (BioTek, Synergy HT, VT, USA). The MIC was defined as the lowest concentration that inhibited the growth of *C. albicans*.

#### 2.7.3. Disk Diffusion Assay

The disk diffusion assay was used to determine the susceptibility of *C. albicans* to PCL-AMB NPs. Briefly, 100 µL of *C. albicans* at a concentration of $1.5\times 10^{8}$ CFU/mL, was used to create a lawn plate on potato dextrose agar. Subsequently, holes were created using a sterile tip, and 100 µL of each test sample (50 µg/mL AMB, 50 µg/mL PCL-AMB NPs, blank PCL NPs, and 0.25% DMSO in potato dextrose broth) were added then incubated at 37 °C for 24 h. Plates were evaluated for their zones of inhibition and reported as diameter in mm.

### 2.8. Cytotoxicity Assay

An MTT assay was used to assess the cytotoxicity of PCL-AMB NPs. Cellular metabolic enzymes (NAD(P)H-dependent cellular oxidoreductase), can reduce MTT to insoluble formazan, resulting in a purple colour, able to be quantified by measuring absorbance at 570 nm. Briefly, 2 × 10^5^ cells/well (HFF-1, ATCC SCRC-1041) in 500 µL of Dulbecco’s Modified Eagle Medium (DMEM, Thermo Fisher Scientific, MA, USA) supplemented with 10% foetal calf serum (FCS, Thermo Fisher Scientific, MA, USA) and 1% (v/v) penicillin/ streptomycin (Thermo Fisher Scientific, MA, USA), were plated in a 48-well plate and allowed to attach for 24 h at 37 °C and 5% CO_2_. Next, media was replenished with the addition of 1x MIC PCL-AMB, and AMB. PCL NPs at the same concentration were also assessed for cytotoxicity, along with untreated wells (tissue culture plate control; TCP) as a baseline measurement. The plates were then incubated for a further 48 h at 37 °C and 5% CO_2._ MTT in DMEM at a concentration of 0.5 mg/mL was then added and incubated for a further 3 h at 37 °C and 5% CO_2._ The media was then removed and 250 µL of Dimethyl sulfoxide was added (DMSO, Sigma-Aldrich, MO, USA), and incubated in the dark for 15 min, on an orbital shaker at 100 rpm. The plate was then measured on a Synergy HTX multimode microplate reader (Biotek, VT, USA) at 570 nm. Viability was calculated by normalizing all samples to the TCP control.

### 2.9. Statistical Data Analysis

Statistical analysis was performed using GraphPad Prism version 9.0.0 for windows (San Diego CA, USA, www.graphpad.com). Cytotoxicity was analysed using One-Way ANOVA followed by Tukey’s multiple comparison post hoc analysis. ImageJ software version 1.52a for windows (National Institute of Health, USA) was used for statistical distribution analysis of particle size. A *p*-value of ≤0.05 was deemed to be statistically significant. All experiments were performed in triplicate.

## 3. Results and Discussion

### 3.1. Nanoparticle Synthesis and Physicochemical Characterization

PCL NPs and PCL-AMB NPs were prepared using the emulsion solvent evaporation method with PVA as the emulsifier. Due to the poor solubility of AMB in DCM, it was necessary to establish an adequate composition of the organic solvent in AMB and PCL. Since AMB and methanol are soluble in DMSO, both were combined at a ratio of 1:4 (DMSO: methanol). The combination was then mixed with DCM at a ratio of 2:3 (DMSO + methanol: DCM) before proceeding with emulsification in PVA. Methanol was employed as a co-solvent to induce a sufficient hydrophobic character to prevent the premature precipitation of PCL upon contact with the aqueous phase [23].

**Figure 1:**
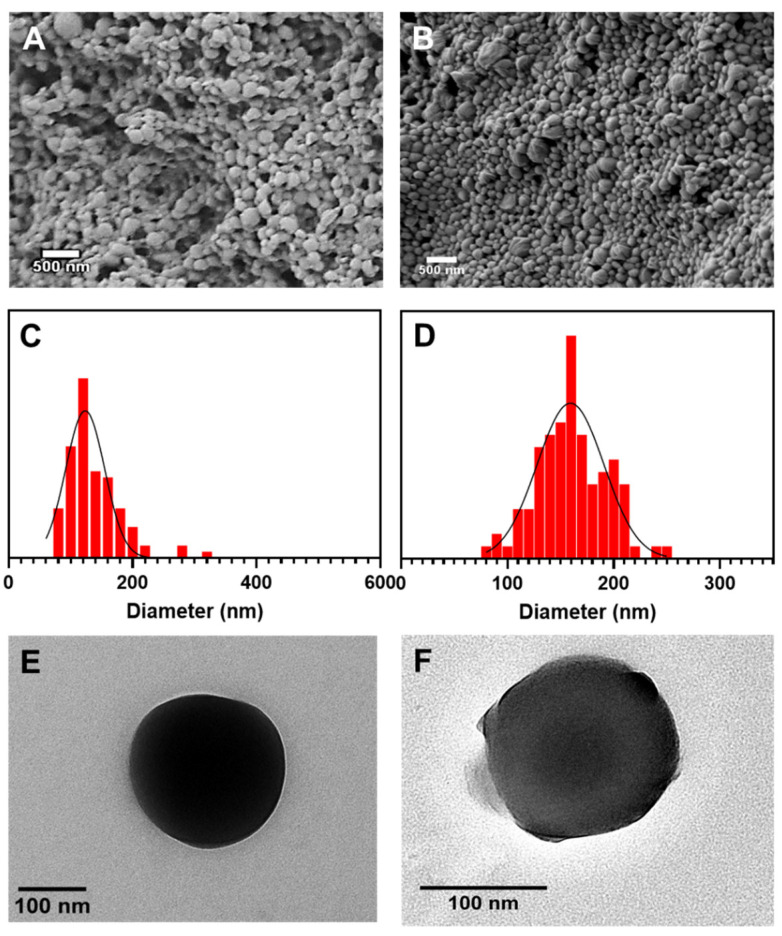
SEM image of PCL NPs (**A**) and PCL-AMB NPs (**B**). Size distribution of PCL NPs (**C**) and PCL-AMB NPs (**D**). TEM images of PCL NPs (**E**) and PCL-AMB NPs (**F**).

**Figure 2:**
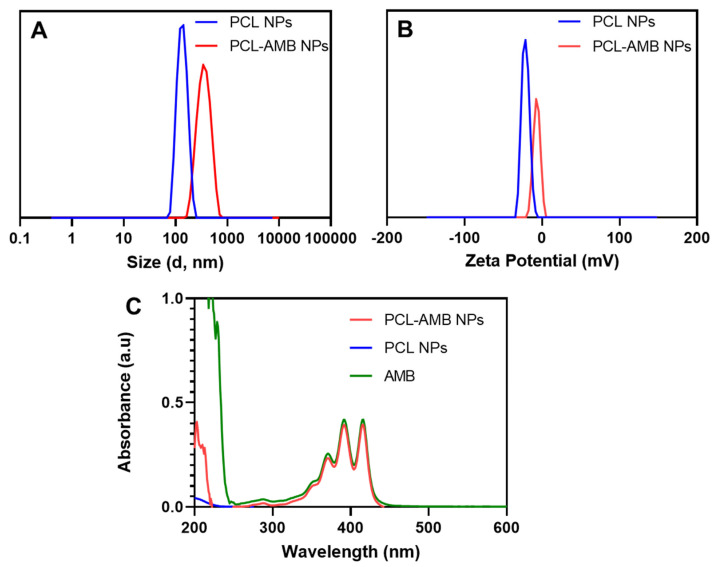
The hydrodynamic diameter of PCL NPs (blue line) and PCL-AMB NPs (red line) (**A**). Zeta potential of PCL NPs (blue line) and PCL-AMB NPs (red line) (**B**). UV-Vis spectra of PCL NPs in MQ water (blue line), AMB in DMSO (green line), and PCL-AMB NPs in DMSO (red line) (**C**).

As shown in Figure 1A, PCL NPs were spherical with an average diameter of 123 ± 31 nm (Figure 1C). The PCL-AMB NPs were also spherical (Figure 1B) with an average diameter of 159 ± 31 nm (Figure 1D). Compared with the TEM micrograph of PCL NPs (Figure 1E), PCL-AMB NP (Figure 1F) reveals that the drug was loaded in the nanoparticle as shown in the core of PCL-AMB NPs. The hydrodynamic diameter of PCL NPs and PCL-AMB NPs was 198.9 ± 41.0 nm and 393.5 ± 104.3 nm, respectively (Figure 2A). The nanomaterial polydispersity index (PDI) values are listed in Table 1, and the values of the formulations signify a narrow nanoscale distribution since PDI values within the range of 0.1 to 0.4 describe moderate polydispersity distribution [16]. The zeta potential of PCL NPs and PCL-AMB NPs were −21.5 ± 4.8 mV and −7.0 ± 4.3 mV, respectively (Figure 2B). The reduced negative zeta potential of PCL-AMB NPs can be attributed to some of the AMB molecules being in proximity to the surface of the nanoparticles. UV-Vis-NIR spectroscopy was used to confirm the loading of AMB in PCL-AMB NPs. The spectra are presented in Figure 2C. No apparent absorption peak was observed in the PCL NPs solution. A typical absorption peak at 415 nm in PCL-AMB NPs is a characteristic of AMB, and occurred in both PCL-AMB NPs and AMB samples, indicating that the drug was successfully loaded in the nanocomposite.

Drug loading and encapsulation efficiency of PCL-AMB NPs was investigated using UV-Vis spectroscopy. This was carried out by correlating the absorbance values with a calibration curve of AMB in 100% DMSO with concentrations of 2, 4, 6, 8, and 10 µg/mL, resulting in an R-squared value of 0.9994 (Figure S1). Drug loading and encapsulation efficiency were found to be 5.9 ± 0.5% and 42.0 ± 3.2%, respectively (Table 2). Furthermore, parameters like nanoparticle size and surface characteristics play fundamental roles in absorption and interaction with biological cells [30,31]. Hence, such crucial parameters need to be controlled and monitored when developing nanocarriers.

### 3.2. Fourier Transform Infrared Spectroscopy (FTIR)

**Figure 3:**
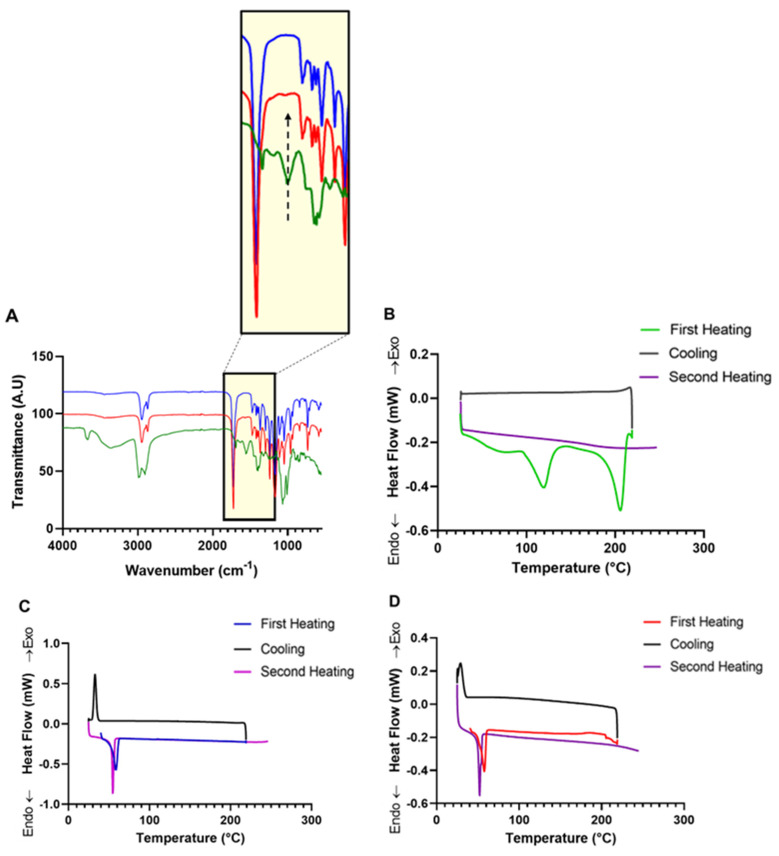
FTIR spectra of AMB (green line), PCL-AMB NPs (red line), and PCL NPs (blue line), black arrow highlights AMB C=C stretch in polyene in the PCL-AMB NPs spectra (**A**). DSC curves of AMB (**B**), PCL NPs (**C**) and PCL-AMB NPs (**D**).

FTIR was performed to study the interaction of AMB in the PCL-AMB NPs nanocomposite and to confirm the incorporation of AMB in PCL-AMB NPs. The FTIR spectra of AMB, PCL NPs, and PCL-AMB NPs are shown in Figure 3A. In PCL NPs, two bands occurred at 2945 cm^−1^ and 2868 cm^−1^ which were attributed to the symmetrical and asymmetric axial deformations of the CH_2_ group, respectively [32]. The most prominent peak observed at 1722 cm^−1^ can be credited to the axial deformation signal of the carbonyl (C=O) present in the ester group. The C–O–C asymmetric stretch occurs at 1238 cm^−1^ while the C–O–C symmetric stretch occurs at 1165 cm^-1^, both indicative of PCL [32,33].

For AMB, the characteristic sharp bands occur at 1690 cm^−1^, 1556 cm^−1,^ and 1008 cm^−1^ which correspond to the C=O stretch, C=C stretch in polyene, and the C–H bend in trans polyene, respectively [34]. Additionally, in AMB, a characteristic peak that represents the C–H stretch in polyene and O–H stretch (strongly H-bonded) occurred at 3359 cm^−1^ [35].

The PCL-AMB NPs exhibited symmetrical and asymmetrical axial deformation of the CH_2_ groups at 2945 cm^−1^ and 2868 cm^−1^, respectively. Additionally, the axial deformation of the C=O group and C–O–C asymmetric stretch occurs at 1722 cm^−1^ and 1238 cm^−1^, respectively. In addition, it can be noted that the C–O–C symmetric stretch in PCL-AMB NPs occurs at 1166 cm^−1^, which indicates a shift from 1165 cm^−1^ when compared to PCL NPs. The C=C stretch in polyene present in AMB, which occurs at 1556 cm^−1^, as highlighted in Figure 3A, can confirm the incorporation of AMB in PCL-AMB NPs.

### 3.3. Differential Scanning Calorimetry (DSC)

DSC measurements of AMB, PCL NPs, and PCL-AMB NPs are shown in Figure 3B–D, respectively. In AMB (Figure 3B), the thermogram shows an endothermic peak at 119.7 °C in the first heating, which can be attributed to the loss of adsorbed water due to the hygroscopic nature of AMB [34]. The thermograph of the first heating cycle of AMB also shows a characteristic endothermic peak at 205.8 °C, which can be attributed to the melting point of AMB [36]. Since AMB starts to degrade over 200 °C without melting, the absence of any thermal effect in the second heating curve indicates the conclusion of degradation [37,38]. PCL NPs and PCL-AMB NPs (Figure 3C,D) showed endothermic melting peaks between 57.7 and 58.5 °C in the first heating, which is indicative of the melting point of PCL [39]. During cooling, PCL NPs and PCL-AMB NPs showed an exothermic peak between 28.9 and 32.8 °C, corresponding to crystallization temperature. In the second heating, the DSC curves of PCL NPs and PCL-AMB NPs showed melting peaks between 52.2 and 54.8 °C. No melting point peak of AMB was observed in the PCL-AMB NPs, suggesting that AMB was dispersed in PCL-AMB NPs and the amount of crystalline AMB present was negligible [40]. In other words, the absence of the AMB peak in PCL-AMB NPs indicates a change in the crystalline state of AMB when loaded in the nanocomposite, suggesting the change in the state of AMB from crystalline to amorphous [30].

### 3.4. In Vitro Drug Release

**Figure 4:**
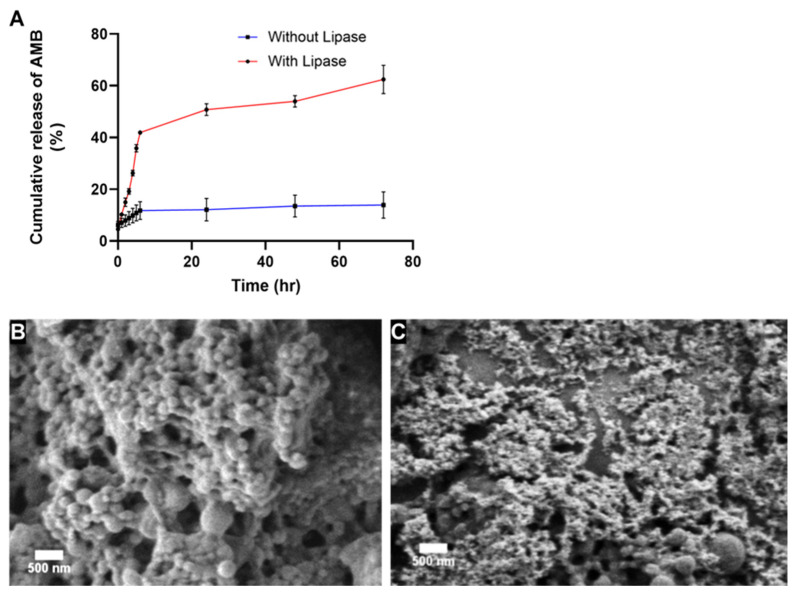
Cumulative release of AMB from PCL-AMB NPs in the presence and absence of lipase (**A**). Surface morphologies of PCL-AMB NPs after 72 h release in the absence of lipase (**B**) and the presence of lipase (**C**). The scale bar represents 500 nm.

The in vitro release of AMB from PCL-AMB NPs was performed in PBS (pH 7.4), representing a common physiological condition, in the presence and absence of lipase at 37 °C for 72 h. The graphical representation of the cumulative release of AMB in percentage versus time is shown in Figure 4A. The results revealed an initial release of about 7% and 10% after 1 h in the absence and presence of lipase, respectively, which may be due to the adsorbed drug on the surface of PCL-AMB NPs. This was followed by a slow release of approximately 11% after 6 h in the absence of lipase. However, in the presence of lipase, a rapid release of approximately 41% occurred after 7 h. As expected, the largest AMB release from PCL-AMB NPs (62%) occurred in the presence of lipase, while in the absence of lipase only 13% of the drug was released after 72 h. The SEM image in Figure 4B shows that after releasing in the absence of lipase, the surface morphology of nanoparticles was not distorted. In comparison, erosion of PCL-AMB NPs after exposure to lipase for 72 h can be easily seen in Figure 4C. The SEM images are in harmony with and support the AMB release data. The slow-release rate in the absence of lipase can be attributed to the high crystallinity and hydrophobicity of the polymer [30,41]. However, when lipase is present, the polymer is degraded within hours providing responsive delivery of the loaded drug [11].

### 3.5. Antifungal Efficacy of PCL-AMB NPs

The MIC and disk diffusion assays were utilized to investigate the antifungal ability of AMB and PCL-AMB NPs against *C. albicans* ATCC 10231. The MIC is defined as the lowest concentration inhibiting the microorganism’s growth [7]. The results show that AMB exhibited an MIC of 0.25 µg/mL (Figure S2B), which is consistent with published studies [42,43]. In comparison, the PCL-AMB NPs exhibited a MIC of 0.06 µg/mL (Figure S2A), which is significantly lower than that of the free drug. This result indicates that the prepared PCL-AMB NPs were more potent against *C. albicans* ATCC 10231. The reason for this finding could be attributed to the increase in adhesion of PCL-AMB NPs to the fungal cell wall, which consequently leads to increased drug penetration into the cells or/and the improved solubility of the drug-loaded nanocomposites when they are degraded by lipase secreted by the fungus [44].

**Figure 5:**
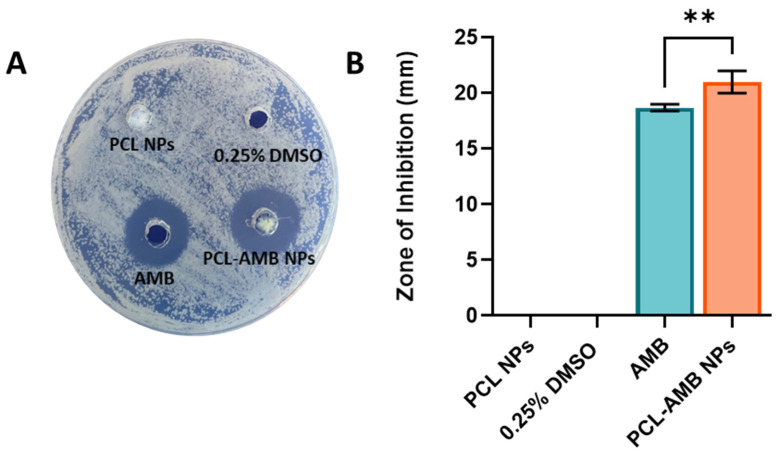
Zone of inhibition for PCL NPs, 0.25% DMSO in MQ water, AMB in 0.25% DMSO, and PCL-AMB NPs in MQ water using *C. albicans* ATCC 10231 (**A**). Zone of inhibition for PCL NPs, 0.25% DMSO in MQ water, free AMB, and PCL-AMB NPs calculated in millimetres (**B**). Bar graphs represent as mean ± SD, n = 3 and ** *p* < 0.01.

The zone of inhibition was determined for PCL NPs, AMB and PCL-AMB NPs using *C. albicans* ATCC 10231 by agar diffusion method which is widely employed for investigating the in vitro antimicrobial activity of various drugs [45] (Figure 5A,B). Since AMB is soluble in DMSO, the latter was used as a co-solvent at a concentration of 0.25% in MQ water. The PCL NPs dispersed in MQ water were used as a control. As seen in Figure 5B, PCL-AMB NPs showed a slightly larger but statistically significant zone of inhibition compared to free AMB at the same concentration (21 ± 1 mm and 18.3 ± 0.3 mm respectively, *p* < 0.01). No zone of inhibition was observed for PCL NPs which is indicative that PCL on its own has no antifungal properties. Additionally, it was found that *C. albicans* was not sensitive to 0.25% DMSO in solution yielding no zone of inhibition, indicating that DMSO did not contribute to the zone of inhibition around the nanocomposite. The results are summarized in Table S1.

### 3.6. Cytotoxicity Assay

**Figure 6:**
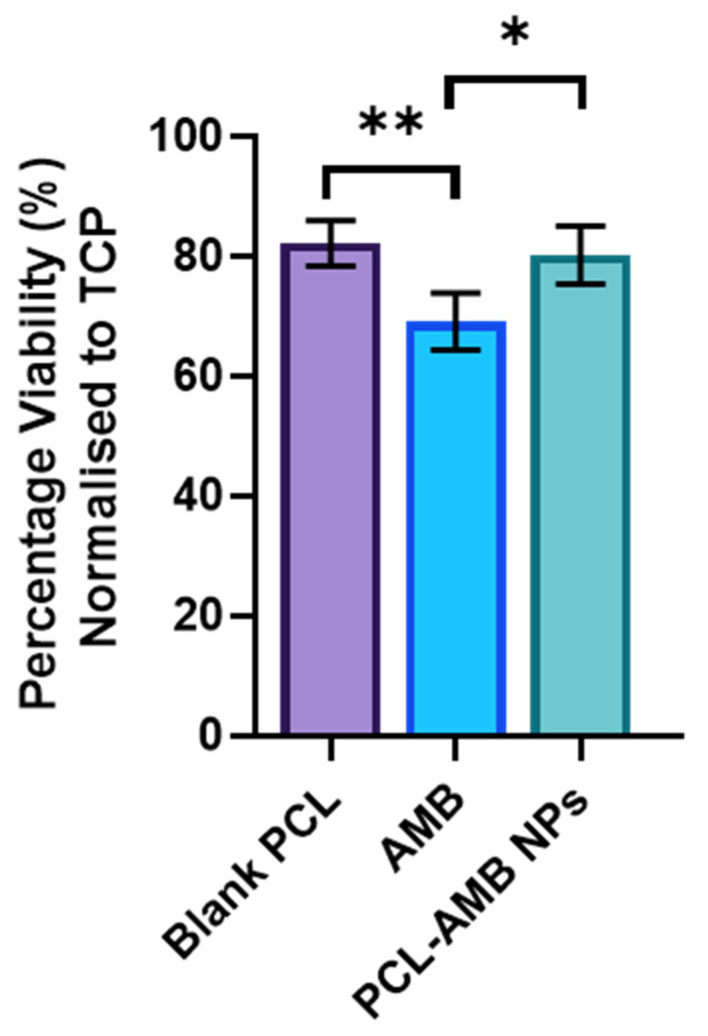
Viability of human dermal fibroblasts treated with PCL NPs, AMB, and PCL-AMP NPs at 1 × MIC, using MTT assay post 48 h treatment normalized to TCP, mean ± SD, n = 3, * *p* < 0.05 and ** *p* < 0.001.

MTT assay was used to assess the cytotoxicity of PCL-AMB NPs against primary human dermal fibroblasts. These cells were chosen as they are imperative for wound healing [46,47]. The results are shown in Figure 6. PCL NPs showed good cell viability (82.1 ± 3.8%) suggesting the PCL vehicle was not cytotoxic. However, there was a significant drop in viability when 1 × MIC of free AMB was incubated with the fibroblasts (69.1 ± 4.7%, *p* = 0.007). Interestingly, the PCL-AMB NPs exhibited similar viability to the blank PCL (80.1 ± 4.9%). These results suggest that loading AMB into PCL-AMB NPs may have increased the safety profile of the antifungal drug.

AMB has been used successfully for the treatment of fungal infections. However, the drug has some limitations related to its poor water solubility. This problem is typically overcome by using high dosages. The side effects include acute and systemic toxicity. On-demand delivery systems have gained research attention due to their capacity to reduce uncontrolled drug release, thereby reducing cytotoxicity to tissues and organs. In this work, we successfully developed a method to encapsulate AMB into PCL nanoparticles for the controlled and targeted, lipase-responsive release of AMB. Our specific target was *C. albicans*, a notoriously stubborn pathogen known to secrete lipase at the site of infection. Furthermore, the nanocomposites designed in this work could have applications beyond *C. albicans* and the concept can readily be adapted to other drugs to target a broader scope of pathogens due to the ubiquity of lipase-expressing pathogens.

## 4. Conclusions

In this work, a lipase-responsive nanomaterial for the on-demand release of AMB was developed. The nanocomposites were prepared by the oil-in-water emulsification method. The results from this study demonstrated the successful encapsulation of AMB in PCL and the responsive release of the drug only in the presence of the enzyme, lipase. Furthermore, the encapsulation in PCL resulted in increased potency of AMB relative to the MIC of the pure drug while improving its cytotoxicity profile. The biocompatible nanocomposites developed in this study have shown promise for future exploitation as a potential candidate that could serve as a delivery vehicle of AMB, providing reduced cytotoxicity and enhanced antifungal efficacy.

## Figures and Tables

**Table 1:** Hydrodynamic and Zeta potential of PCL NPs and PCL-AMB NPs.

Sample	Hydrodynamic Diameter (nm)	PDI	Zeta Potential (mV)
PCL NPs	198.9 ± 41.1	0.25 ± 0.04	−21.5 ± 4.8
PCL-AMB NPs	393.5 ± 104.3	0.33 ± 0.1	−7.0 ± 4.3

**Table 2:** Drug loading and encapsulation efficiency of PCL-AMB NPs.

Sample	Yield (%)	Drug Loading (%)	Encapsulation Efficiency (%)
PCL-AMB NPs	46.9 ± 4.4	5.9 ± 0.5	42.0 ± 3.2

## Data Availability

Data can be available upon request from the authors.

## Supplementary Materials

The following supporting information can be downloaded at: https://www.mdpi.com/article/10.3390/nano13010155/s1, Figure S1: Calibration curve of AMB in DMSO; Figure S2: MIC of AMB (A) and PCL-AMB NPs (B); Table S1: Summary of results for the disk diffusion assay.
