# Supplementary material for: Lipase-Responsive Amphotericin B Loaded PCL Nanoparticles for Antifungal Therapies
Source: Nanomaterials (Basel). 2022 Dec 29;13(1):155. doi: 10.3390/nano13010155 (PMC9823996; doi:10.3390/nano13010155)
Supplement: Supplementary file 1 [file nanomaterials-13-00155-s001.zip › nanomaterials-2112121-supplementary.pdf]

SUPPORTING INFORMATION

Lipase-Responsive Amphotericin B Loaded PCL Nanoparticles for Antifungal Therapies

Evelyn Osehontue Uroro [1], Richard Bright [2,*], Andrew Hayles [2] and Krasimir Vasilev [1,2,*]

[1] UniSA STEM, University of South Australia, Mawson Lakes, South Australia, SA 5095, Australia
[2] College of Medicine and Public Health, Flinders University, Bedford Park, SA 5042, Australia
* Correspondence: richard.bright@flinders.edu.au (R.B.); krasimir.vasilev@flinders.edu.au (K.V.)

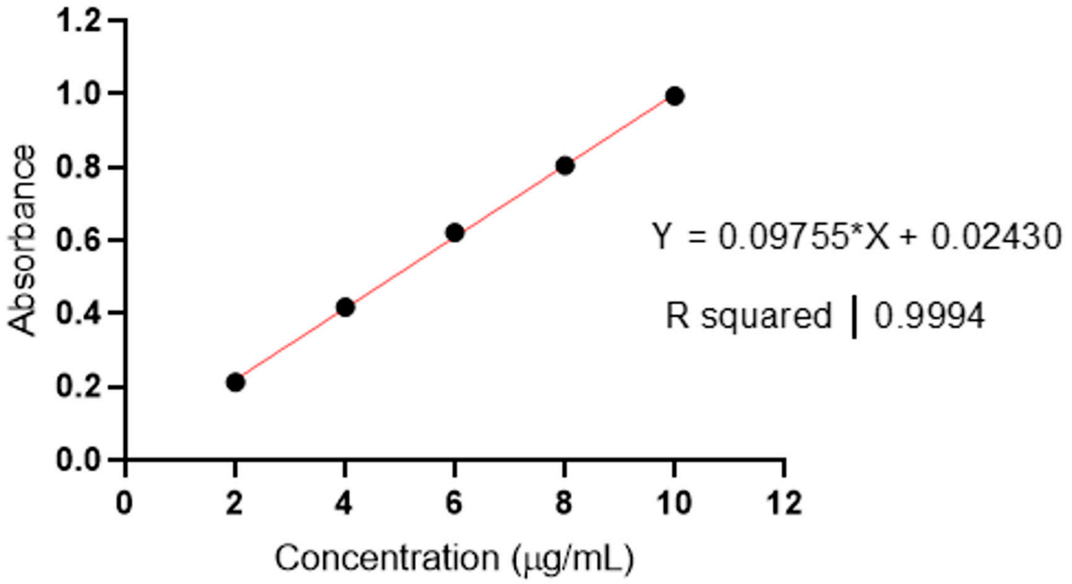

Figure S1. Calibration curve of AMB in DMSO.

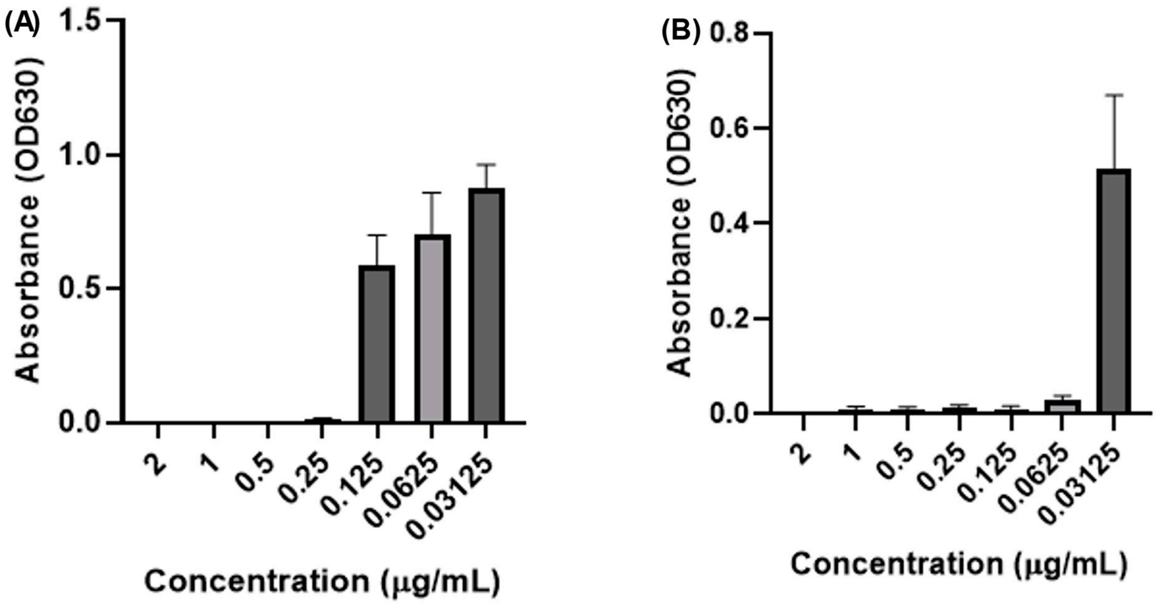

Figure S2. MIC of AMB (**A**) and PCL-AMB NPs (**B**).

Table S1: Summary of results for the disk diffusion assay.

Sample	Zone of Inhibition (mm)
PCL NPs	0
0.25% DMSO in MQ water	0
AMB in 0.25% DMSO	18.7 ± 0.3
PCL-AMB NPs	21.0 ± 1.0
